# Molecular Mechanism of Xixin-Ganjiang Herb Pair Treating Chronic Obstructive Pulmonary Disease-Integrated Network Pharmacology and Molecular Docking

**DOI:** 10.1155/2021/5532009

**Published:** 2021-06-10

**Authors:** Ping Huang, Tao Huang, Deshun Li, Lintao Han, Zhenxiang Zhou, Fang Huang, Jingjing Li, Jiajia Wu, Yan Ye, Qiong Wang, Bailu Duan

**Affiliations:** ^1^College of Basic Medicine, Hubei University of Chinese Medicine, Wuhan 430065, China; ^2^Wuhan Red Cross Hospital, Wuhan 430065, China; ^3^Pharmacy School, Hubei University of Chinese Medicine, Wuhan 430065, China

## Abstract

**Background:**

Chronic obstructive pulmonary disease (COPD) is characterized by high morbidity, disability, and mortality, which seriously threatens human life and health. Xixin and Ganjiang are classic herb pairs of Zhongjing Zhang, which are often used to treat COPD in China. However, the substance basis and mechanism of action of Xixin-Ganjiang herb pair (XGHP) in the treatment of COPD remain unclear.

**Methods:**

On the website of TCMSP and the DrugBank, effective compounds and targets of XGHP were found. COPD targets were obtained from GeneCards, DisGeNET, and GEO gene chips. Intersecting these databases resulted in a library of drug targets for COPD. Then, intersection targets were used for protein-protein interaction (PPI) and pathway enrichment analysis. Finally, the binding activity between compounds and core genes was evaluated by molecular docking to verify the expression level of PTGS2 and PPARG in rats.

**Results:**

Twelve effective compounds and 104 core genes were found in the intersection library, and kaempferol, sesamin, *β*-sitosterol, PTGS2, and PPARG were particularly prominent in the network analysis. A total of 113 pathways were obtained and enrichment of the TNF signaling pathway, IL-17 signaling pathway, and C-type lectin receptor signaling pathway was particularly obvious. Molecular docking indicated that kaempferol, sesamin, and *β*-sitosterol were closely related to PTGS2 and PPARG and were superior to aminophylline. Key compounds in XGHP could restrict the expression of PTGS2 in the lung tissues of COPD rats and promote the expression of PPARG.

**Conclusion:**

Inhibition of the expression of inflammatory factor PTGS2 and promotion of the expression of PPARG may be an effective target of XGHP in the treatment of COPD.

## 1. Introduction

Chronic obstructive pulmonary disease (COPD) is a progressive chronic respiratory disease with a high morbidity and mortality [1, 2]. Airflow restriction, chronic bronchitis, and chronic airway obstruction may decline pulmonary function in a progressive and irreversible manner. Data published by the World Health Organization (WHO) showed that nearly 3 million people worldwide die from COPD each year [3]. Smoking, environmental effects, and occupational exposure are the main causes of COPD. The pathological changes of COPD are mainly immune reactions occurring in the central airway, small airway, and the alveolar space. The protease-antiprotease hypothesis, immune mechanism, oxidation-antioxidant balance, and systemic inflammation have all been considered to be related to the pathogenesis of COPD [4]. Takeuchi et al. suggested that the development of emphysema was related to these types of immune inflammation, which increase the production of mucus and disrupt the lungs' gas-exchange surfaces [5]. Moreover, chronic inflammation is key in COPD development, and abnormal distribution of neutrophils, macrophages, and lymphocytes has been found in the small airways of patients with COPD [6, 7]. So far, no drugs for COPD have been proven to alter the long-term decline in pulmonary function, and bronchodilators are the primary treatment for COPD. By 2030, COPD is projected to be the third leading killer of human life and health [8]. The WHO has designated the Wednesday in the third week of November as World COPD Day to raise awareness and strengthen the prevention and treatment of COPD.

Historically, Traditional Chinese Medicine (TCM) has achieved a great curative effect in the treatment of various diseases, because of its integrated conditioning ability and few adverse reactions. In the process of treating diseases in TCM, different compatibility between herbs can play different therapeutic roles, even if medicine can contain dozens of herbs. Herb pair is the simplest compatibility, which contains only two herbs [9]. XGHP was derived from *ShangHanZaBingLun*, written by Zhongjing Zhang in the classic prescriptions of Xiaoqinglong decoction, Linggan Wuwei Jiangxin decoction, and other medications. XGHP is widely and effectively used to treat COPD. Xiaoqinglong decoction, which contains XGHP, has been shown to inhibit excessive airway mucus secretion, reduce airway obstruction, and improve lung ventilation function [10]. Linggan Wuwei Jiangxin decoction could regulate the content of MUC5AC in rats and regulate the secretion of airway fluid to treat lung disease with a Cold Syndrome [11, 12]. However, the effective substance and underlying mechanism of action of the therapeutic effect of XGHP have not yet been identified, which restricts its clinical application and development.

Network pharmacology has been widely used to explore novel drugs and repurpose existing drugs [13]. Through the establishment of a “compound-gene-disease” network, it was more effective to reveal the regulation principle of small molecules with high throughput, compared with the contemporary “one target, one drug” mode [14]. Because of the complex composition in herbs and the multiple targets in diseases, network pharmacology has become an effective tool in predicting novel drug targets and mining the material basis of the TCM system.

**Figure 1:**
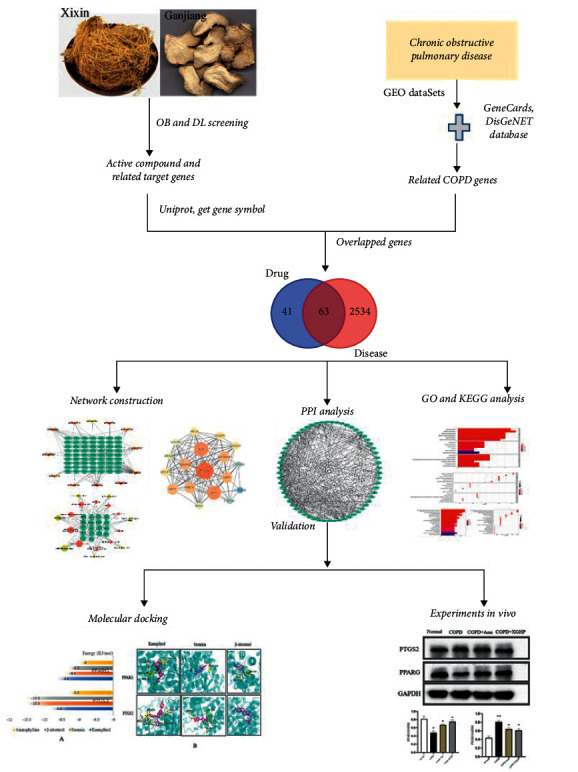
Flowchart of mechanism exploration of XGHP treatment for COPD.

In the current study, the core components and targets of XGHP were be predicted by network pharmacology and verified by molecular docking and in vivo verification to explore the active substances and pathways of the herb pair. The working flowchart is presented in Figure 1.

## 2. Materials and Methods

### 2.1. Collection of the Small Molecule of XGHP and Screening

In this study, we followed the methods of Duan et al. 2020 [15] to obtain chemical compounds in Xixin (XX) and Ganjiang (GJ) from TCMSP (http://tcmspw.com/tcmsp.php) [16]. Based on literature reports, ingredients that met OB ≥ 30% and DL ≥ 0.18 were screened out as the main active ingredients of herbs [17]. In addition, compounds were supplemented by literature retrieval and data mining. Potential protein targets of XGHP main compounds were corrected through UniProt (https://www.uniprot.org/) databases.

### 2.2. Establishment of Database for Targets Associated with COPD

Microarray data of differentially expressed RNA from alveolar macrophages in normal and COPD groups were downloaded from the GEO database (https://www.ncbi.nlm.nih.gov/geo/), series: GSE130928, platforms: GPL570-55999. The Limma package in the Bioconductor platform (https://www.bioconductor.org/) and R3.6.3 software were used for chip analysis. Genes with *P* < 0.05 and log2 (FC) < > 1 or log2 (FC) < −1 were selected, and these genes were thought to be significantly differentially expressed associated with COPD. In addition, disease genes from the Human Gene Database (https://www.genecards.org) and DisGeNET (https://www.disgenet.org) databases were combined, duplicate disease targets were eliminated, and a COPD disease targets database was established.

### 2.3. Creation of XGHP-COPD Network Diagrams

Next, the VEEN tool (http://bioinformatics.psb.ugent.be/cgi-bin/liste/Venn/calculate_venn.htpl) was used to intersect the effective drug targets and disease targets, and the core targets of XGHP-COPD were obtained. The core targets were then uploaded into STRING (https://stringdb.org/cgi/input.pl), which provided information on the interaction of proteins [18] to construct the protein-protein interaction (PPI) network. Next, we used Cytoscape 3.7.2 (https://www.cytoscape.org/) and its attachments CytoNCA inside the parameters of the Degree of Centricity (DC), Closeness Centrality (CC), and Betweenness Centrality (BC) to further filter out core targets [19].

### 2.4. Analyses of Enrichment Pathway

The ClusterProfile package [20] was downloaded from the Bioconductor website and applied to R 3.6.3 software to obtain Gene Ontology (GO) and Kyoto Encyclopedia of Genes and Genomes (KEGG) enrichment information of overlapping targets. Subsequently, related “histograms” and “bubble graphs” were established.

## 3. Validation

### 3.1. Molecular Docking

To further examine the predictive power of our previous network pharmacology, molecular docking techniques were used to determine the ability of the screened active ingredients to bind to these proteins. The PDB website (http://www1.rcsb.org/) was used to download the protein structure, while its 3D form was available on the PubChem website (https://pubchem.ncbi.nlm.nih.gov/). Furthermore, PyMol software removed water molecules and small molecule ligands, and the receptor and ligand were hydrogenated by AutoDock.

### 3.2. Efficacy Evaluation of XGHP in a COPD Rat Model

#### 3.2.1. Reagents

Xixin and Ganjiang were purchased from the TCM Pharmacy of Wuhan Hospital of Traditional Chinese Medicine (Wuhan, China). According to the ratio of 1 : 1, the amount of crude drug per unit body weight of rats was obtained according to the amount of crude drug per unit body weight of human, enlarged by 10 times to weigh the restorative materials and prepare the Xixin-Ganjiang decoction [21]. Water was added to boil twice for 30 min and then filtered and concentrated at 60°C in a constant temperature water bath. Finally, the XGHP decocted concentration was 2.7 g/ml, which was stored in the refrigerator for further use. The cigarettes were Yellow Crane Tower (Wuhan, China). Lipopolysaccharide was purchased from Sigma (St. Louis, MO, USA). Chloral hydrate was purchased from Tianjin Damao Chemical Reagent Factory (Tianjin, China). Primary antibodies: anti-PTGS2(#12282) was purchased from CST (Massachusetts, USA), anti-PPARG (GB11164), and anti-GAPDH (GB11002) were purchased from Servicebio (Wuhan, China). Secondary antibody: HRP-labeled Goat Anti-Rabbit lgG (H+L) was purchased from Servicebio (Wuhan, China).

#### 3.2.2. Animals

Adult Sprague Dawley (SD) male rats (220 ∼ 250 g; aged 6–8 weeks; males) were recruited from Hubei Experimental Animal Research Center (Wuhan, China) and were fed in a specific pathogen-free (SPF) laboratory of Hubei University of TCM (Wuhan, China) at room temperature, a humidity of 50 ± 10%, and 12 h day and night light cycle. Rats were fed standard feed pellets and had free access to water. The Animal Ethics Committee of Hubei University of Chinese Medicine approved the study protocol.

#### 3.2.3. Induction and Administration of COPD

Rats were randomly divided into four groups: normal group, COPD group, COPD + XGHP group, and a COPD + aminophylline (Ami) group (10 rats per group). Rats were injected with LPS (200 *μ*l/injection) on the first and 14th day, respectively. Rats, except for rats in the normal group, were exposed to 14 cigarettes of smoke for 30 minutes at 4-hour intervals in a smoke box from the second day to the 30th day (except for day 14), frozen at 0°C for 1h/d, and given a mixture of ice and water by gacaged (1ml/100g) to replicate the model of cold drink accumulation of lung syndrome. Normal group rats were given control treatment. From day 16, rats in the normal group and COPD group were gavaged with 1 ml/100 g saline once a day, and rats in the COPD + XGHP group were gavaged with 1 ml/100 g traditional medicine decoction once a day. In the COPD + Ami group, 1 ml/100 g aminophylline solution (5 g/l) was gavaged once a day for 14 days.

At the end of the experiment, lung tissues were removed under chloral hydrate anesthesia and labeled and stored in liquid nitrogen for Western blot analysis.

#### 3.2.4. Western Blot Analysis

Lung tissues were weighed, minced with steel balls, and dissolved in 100 mmol/L PMSF radioimmunoprecipitation (RIPA) buffer. The homogenate was then centrifuged at 4°C and 12,000 rpm for 10 min. Then, the supernatant was collected and 5*∗* loading buffer was added proportionally. The solution was sterilized at 100°C heating water for 15 min. Finally, it was cooled to room temperature and divided into Eppendorf tubes. The proteins were isolated and separated on a 12% SDS-PAGE and electrophoretically transferred onto PVDF membranes. Membranes were blocked with 5% skim milk (TBST −0.1% Tween-20, TBST) for 1 hour at room temperature and incubated with primary antibodies at 1 : 1000 dilution at 4°C overnight. Next, membranes were washed for 3 times with TBST solution (5 min for each), followed by incubation with HRP-labeled Goat Anti-Rabbit lgG (*H* + *L*) for 30 min. Then, membranes were rinsed 3 times with TBST solution and the immunoreactive zone was observed with an enhanced chemiluminescence (ECL) reagent kit. The density of each band was analyzed by using Bio-Rad Quantity One software. GAPDH was selected as an internal reference for semiquantitative analysis.

#### 3.2.5. Statistical Analysis

Statistical analysis and mapping were performed using GraphPad Prism 8 software (San Diego, CA, USA). Differences between groups were statistically compared by unpaired *t*-test. One-way analysis of variance was used for multiple comparisons. *P* < 0.05 was considered statistically significant.

## 4. Results

### 4.1. Screening of Active Components and Targets of XGHP

Xixin (XX) contains 192 components and Ganjiang (GJ) 148 components. All active ingredients met the screening rules, OB ≥ 30% and DL ≥ 0.18. After screening, 12 core active compounds of XGHP were identified (Table 1), including 8 compounds in XX and 5 compounds in GJ. MOL002501 is a shared compound. A total of 428 potential targets were found for XGHP from TCMSP and DrugBank, and the UniProt database was used for standardization. Finally, 104 potential drug targets were obtained.

### 4.2. Targets of XGHP against COPD

**Figure 2:**
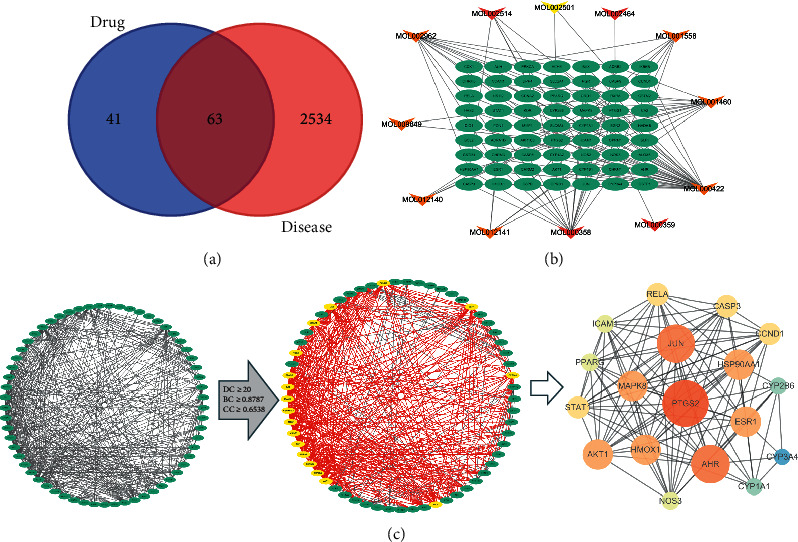
(a) Common targets between XGHP targets and COPD targets; (b) components-targets network. Orange nodes represent Xixin. Red nodes represent Ganjiang, and yellow nodes represent Xixin and Ganjiang, while green nodes represent targets of XGHP. (c) Topology filtering process of PPI network. 61 XGHP-COPD composite targets were screened by DC ≥ 20, BC ≥ 0.8787, and CC ≥ 0.6538, and finally, the PPI network of 18 hub nodes was obtained.

Using the GEO database microarray analysis (series: GSE130928, platforms: GPL570-55999), 700 differentially expressed genes were identified that were related to COPD (Table S1). Next, 2597 disease-related targets were identified by integrating the Human Gene Database, DisGeNET Database Disease Targets, and GEO databases after eliminating duplicates. By intersecting XGHP active component targets with COPD disease targets, 63 XGHP-COPD composite targets were obtained (Figure 2(a)). Subsequently, the “components-targets” network of XGHP-COPD was established (Figure 2(b)). Kaempferol, *β*-sitosterol, and sesamin may be the central core components of XGHP-COPD.

### 4.3. PPI Network of Target Genes

The PPI network graph was obtained by importing 63 compound targets into STRING and by removing two disconnected points. There were 61 nodes and 442 edges, the average number of nodes was 14, and the average local clustering coefficient was 0.579. The TSV data was downloaded and imported into Cytoscape 3.7.2 to show the protein interaction network. Then, according to the three parameters DC, BC, and CC, the median target was selected as the key target for construction of the XGHP anti-COPD hub node. The screening criteria were DC ≧ 20, CC ≧ 0.6538, and BC ≧ 0.8787. The results included 18 hub nodes and 112 edges [22]. Finally, we adjusted the node properties in the network according to the degree value as follows: the larger the degree value of the target, the larger the area of the node (low values to dark colors) (Figure 2(c)).

### 4.4. Analyses of the Enrichment Pathway

**Figure 3:**
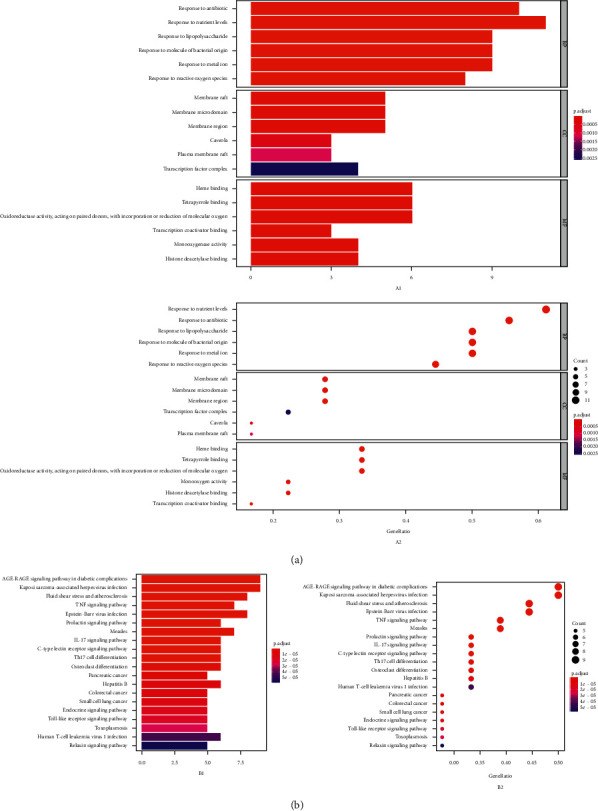
(a) A1 and A2: functional enrichment analysis based on GO database. (b) B1 and B2: pathway enrichment analysis based on KEGG database.

**Figure 4:**
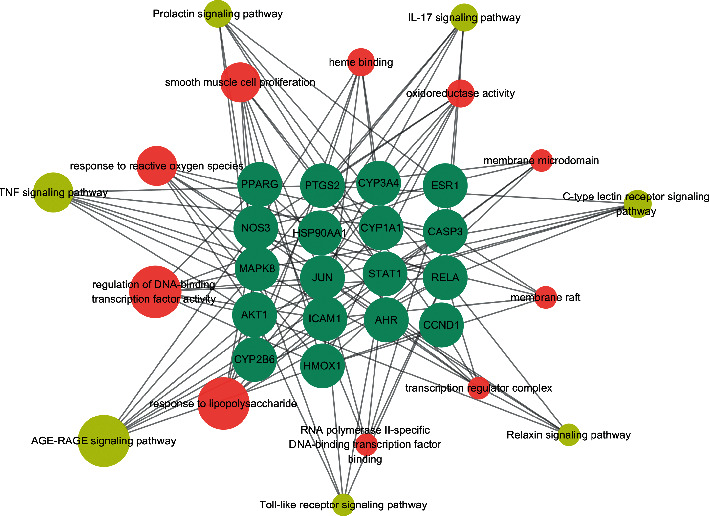
Networks for “Targets-biological enrichment-pathways.” The genes of XGHP-COPD showed up as green. The GO biological information showed up as pink. The related pathways showed up as yellow.

In this study, we mainly found the life activities related to 18 hub targets in cellular composition, molecular function, and biological process. As the biological process was 760, XGHP treatment of COPD mainly involved the response to antibiotics, reactive oxygen species, and lipopolysaccharide, such as regulation of smooth muscle cell proliferation (GO:0048660), regulation of DNA-binding transcription factor activity (GO:0051090), reactive oxygen species biosynthetic process (GO:1903409), and nitric oxide biosynthetic process (GO:0006809). The cellular composition was 8, which mainly involved membrane region (GO:0098589), transcription factor complex (GO:0005667), and nuclear chromatin (GO:0000790). The MF was 73. This mainly involved nuclear transcription, oxidoreductase activity, and heme binding, such as RNA polymerase II transcription factor binding (GO:0001085), nuclear receptor activity (GO:0004879), and protein phosphatase binding (GO:0019903). Information on the top six enrichments in cellular composition, molecular function, and biological process were selected to create a bubble chart and bar chart. Moreover, the main signaling pathway of XGHP treatment for COPD was identified and analyzed by KEGG enrichment. KEGG pathway analysis returned 113 items, including the AGE-RAGE signaling pathway (hsa04933), TNF signaling pathway (hsa04668), and IL-17 signaling pathway (hsa04657) (Figure 3). The first 20 remarkable pathways were selected and presented in Table S2. Then, the network of the “Targets-biological enrichment-pathways network” (Figure 4) was established.

## 5. Validation

### 5.1. Molecular Docking

**Figure 5:**
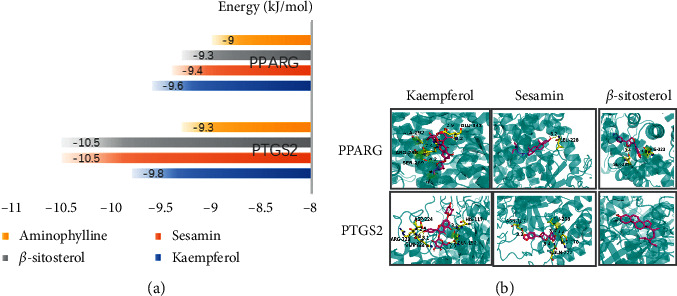
(a) Binding results of hub ingredients of XGHP and aminophylline with proteins. (b) Molecular docking model diagram of key pharmacodynamic substances-core targets.

In molecular docking, the less the binding energy between the ingredient and proteins is, the more likely the docking is to occur. In general, a compound with a binding fraction has a better binding activity with the target. In this study, the 3 active components of degree, including kaempferol, sesamin, and *β*-sitosterol, were selected as candidate docking components. Moreover, PTGS2 and PPARG were chosen as the candidate targets, and aminophylline was selected as the positive drug for molecular docking. The results are presented in Figure 5. It can be concluded that kaempferol, sesamin, and *β*-sitosterol have a higher score than aminophylline for PTGS2 and PPARG, and it was suggested that the active components in XGHP closely bound to the predicted target, which may be the main basis for the treatment of COPD.

### 5.2. Western Blot Analysis

**Figure 6:**
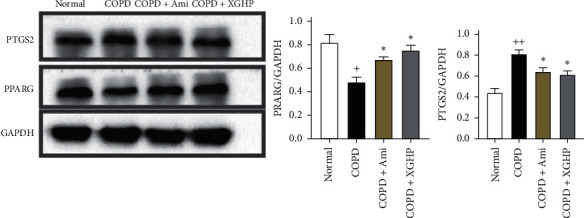
Effect of XGHP on PTGS2 and PPARG in lung in each group rats. ^++^*P* < 0.01 versus the normal group; ^*∗*^*P* < 0.05 versus the COPD group. The value is expressed as mean ± SEM.

Based on network pharmacological analysis, PTGS2 and PPARG were selected for experimental verification. The results of Western blot analysis showed that that the PPARG protein content was significantly different between the normal group and the COPD group (*P* < 0.05). Treatment with XGHP or aminophylline showed that PPARG expression significantly increased in the COPD + XGHP group or COPD + Ami group compared with the COPD group. The protein content of PTGS2 was markedly different between the COPD group and the normal group (*P* < 0.01). Furthermore, treatment with XGHP/aminophylline showed that PTGS2 expression significantly decreased in the COPD + XGHP group or COPD + Ami group compared with the COPD group (*P* < 0.05) (Figure 6).

## 6. Discussion

In the current study, network pharmacological was used to predict the substance basis and mechanism of action of XGHP on COPD. A total of 12 active ingredients and 104 corresponding core proteins were identified, and the active ingredients were closely linked to targets in the PPI network diagram. The KEGG signaling pathway also revealed that these targets were directly or indirectly involved in various inflammatory responses, including TNF signaling and IL-17 signaling. Moreover, molecular docking experiments revealed that the hub components in XGHP combined with the core targeted better than aminophylline. In the study, the expression of related inflammatory targets decreased or increased with the effect of XGHP, thereby indicating that Chinese medicine can effectively reduce airway inflammation.

Inflammation is a common clinical pathological process, which can occur in tissues and organs in various parts of the body. In general, inflammation is a type of antidisease response of the body, which is conducive to the recovery of balance of the body. However, under certain conditions, some beneficial factors in the inflammatory response can be transformed in the opposite direction and become harmful factors to the body. A growing body of evidence has shown that Chinese herbs can suppress inflammation. Mucus hypersecretion (MH) in the airway is always accompanied by COPD, which is an independent risk factor affecting COPD disease change and prognosis [23]. Inhibition of several inflammatory pathways to reduce the inflammatory response can effectively reduce MH [24]. The efficacy and safety of XGHP in the treatment of COPD have been widely recognized. In this study, we focused on identifying the substance basis and pharmacological pathway of the role of XGHP.

In this study, kaempferol, *β*-sitosterol, and sesamin may be the important ingredients of XGHP in COPD treatment. Kaempferol, one of the essential ingredients, was found in 12 active ingredients of XGHP with 39 targets. In many studies, it has been shown that kaempferol can reduce various types of inflammation induced by the lipopolysaccharide (LPS), such as colitis, neuroinflammation, and rheumatoid arthritis [25–27]. LPS-induced inflammation is often used as one of the methods to establish COPD models. The mechanism of action of kaempferol is to inhibit the nuclear translocation of signal transducer and activator of transcription [28, 29], thereby inhibiting the expression of PTGS2 and activating the NF-*κ* B signaling pathway [30, 31]. *β*-sitosterol, a sterol commonly found in herbs, was used to treat pathological changes in lung tissue and prevent airway inflammation [32] and also showed anti-inflammatory effects in macrophages, microglia, and joint tissues [33–35]. Moreover, *β*-sitosterol could reduce the LPS-induced expression of PTGS2 and exert anti-inflammatory and analgesic effects [36]. Kaempferol, *β*-sitosterol, and sesamin can enhance insulin resistance, protect myocardial cells from injury, and prevent hyperlipidemia by increasing the expression of PPARG [37, 38]. Sesamin also reduced the overexpression of PTGS2 by regulating the JNK and p38 MAP kinase pathways [39]. *In vitro* experiments showed that sesamin could significantly inhibit the expression of PTGS2 in a dose-dependent manner [40, 41]. In the literature, these three active ingredients have shown significant anti-inflammatory effects, which indicated that anti-inflammatory effects are an important drug basis of XGHP and an essential link in the treatment of COPD.

A significant decrease in the number of various inflammatory cells in the administration group was found to be related to the inhibition of PTGS2 expression [42]. Wang et al. demonstrated that the inhibition of PTGS2 could regulate the expression of aquaporin-1 and alleviate lung injury [43]. Furthermore, PTGS2 was directly enriched in related inflammation pathways, such as the TNF signaling pathway and the IL-17 signaling pathway, and in small cell lung cancer. Moreover, PTGS2 and PPARG were directly connected in the PPI network, thus suggesting that XGHP exerts an anti-inflammatory effect through a multitarget and multipathway combination rather than single pathway. PPARG, a subtype of PPARs, is a nuclear receptor family member. PPARG and its ligands play a vital role in lipid and glucose metabolism. Cho et al. demonstrated that rosiglitazone (PPARG agonist) could protect lung tissues by inhibiting NOx/ROS/C-SRC/PYK2/Akt-dependent activation of NRF2, either by inducing HO-1 expression or by inhibiting NF-*κ*B expression, thereby further inhibiting activation of TLR2/NLRP3 inflammasomes [44]. In addition, a variety of *in vitro* experiments have demonstrated that PPARG can regulate mucinous proteins and inflammatory cytokines in lung tissue, thereby exaggerating the expression of airway hyperresponsiveness, inflammation, and cytokines [45, 46]. Taken together, these results indicated that PPARG can improve pulmonary inflammation through various signaling pathways.

## 7. Conclusion

In conclusion, we predicted and verified that XGHP is beneficial to restrain the expression of PTGS2 in lung tissue and promote the expression of PPARG through various inflammation-related pathways, thus treating COPD and restoring lung function. Although the results reveal that XGHP may influence COPD through inflammatory responses and LPS and partially explain the relevant targets of anti-COPD, we still need to further verify the relevant pathways involved and explore the pharmacological mechanism of COPD treatment.

## Figures and Tables

**Table 1:** A list of 12 active compounds.

MOL	Compound	OB	DL	Degree	Herb
MOL012140	4, 9-Dimethoxy-1-vinyl-*β*-carboline	65.30	0.19	4	XX
MOL012141	Caribine	37.06	0.83	5	XX
MOL001460	Cryptopine	78.74	0.72	13	XX
MOL001558	Sesamin	56.55	0.83	7	XX
MOL002501	[(1S)-3-[(E)-but-2-enyl]-2-Methyl-4-oxo-1-cyclopent-2-enyl] (1R, 3R)-3-[(E)-3-methoxy-2-methyl-3-oxoprop-1-enyl]-2, 2-dimethylcyclopropane-1-carboxylate	62.52	0.31	1	XX&GJ
MOL002962	(3S)-7-Hydroxy-3-(2, 3, 4-trimethoxyphenyl)chroman-4-one	48.23	0.33	12	XX
MOL000422	Kaempferol	41.88	0.24	39	XX
MOL009849	ZINC05223929	31.57	0.83	2	XX
MOL002464	1-Monolinolein	37.18	0.30	1	GJ
MOL002514	Sexangularetin	62.82	0.31	5	GJ
MOL000358	Beta-sitosterol	36.91	0.75	19	GJ
MOL000359	Sitosterol	36.91	0.75	1	GJ

## Data Availability

The data used to support the findings of this study are available from the corresponding author upon request.

## Abbreviations

COPD: Chronic obstructive pulmonary disease
XGHP: Xixin-Ganjiang herb pair
XX: Xixin (Asarum)
GJ: Ganjiang (Zingiberis Rhizoma)
GEO: Gene Expression Omnibus
PPI: Protein-protein interaction
GO: Gene ontology
KEGG: Kyoto Encyclopedia of Genes and Genomes
TCM: Traditional Chinese Medicine
MUC5AC: Mucin 5AC
OB: Oral bioavailability
DL: Drug-likeness
FC: Fold change
DC: Degree of centricity
CC: Closeness centrality
BC: Betweenness centrality
SD: Sprague Dawley
Ami: Aminophylline
LPS: Lipopolysaccharide
PDB: Protein Data Bank
PMSF: Phenyl methyl sulfonyl fluoride
SDS-PAGE: Polyacrylamide gel electrophoresis
PVDF: Polyvinylidene fluoride
TBST: Tris buffer solution Tween
WB: Western blot
HO-1: Heme oxygenase.

## Supplementary Materials

Supplementary Materials Table S1: differentially expressed genes in GEO chip. Table S2: top 20 KEGG pathway analysis.
