# Supplementary material for: Molecular Mechanism of Xixin-Ganjiang Herb Pair Treating Chronic Obstructive Pulmonary Disease-Integrated Network Pharmacology and Molecular Docking
Source: Evid Based Complement Alternat Med. 2021 Jun 10;2021:5532009. doi: 10.1155/2021/5532009 (PMC8211495; doi:10.1155/2021/5532009)
Supplement: Supplementary Materials — Table S1: differentially expressed genes in GEO chip. Table S2: top 20 KEGG pathway analysis. [file 5532009.f1.zip › Table S1 (1).docx]

| NO. | Pathway | Gene number | | Genes |
| --- | --- | --- | --- | --- |
| hsa04933 | AGE-RAGE signaling pathway in diabetic complication | 9 | STAT1/RELA/NOS3/MAPK8/JUN/ICAM1/CCND1/CASP3/AKT1 | |
| hsa05167 | Kaposi sarcoma-associated herpesvirus infection | 9 | STAT1/RELA/PTGS2/MAPK8/JUN/ICAM1/CCND1/CASP3/AKT1 | |
| hsa05418 | Fluid shear stress and atherosclerosis | 8 | RELA/NOS3/MAPK8/JUN/ICAM1/HSP90AA1/HMOX1/AKT1 | |
| hsa04668 | TNF signaling pathway | 7 | RELA/PTGS2/MAPK8/JUN/ICAM1/CASP3/AKT1 | |
| hsa05169 | Epstein-Barr virus infection | 8 | STAT1/RELA/MAPK8/JUN/ICAM1/CCND1/CASP3/AKT1 | |
| hsa04917 | Prolactin signaling pathway | 6 | STAT1/RELA/MAPK8/ESR1/CCND1/AKT1 | |
| hsa05162 | Measles | 7 | STAT1/RELA/MAPK8/JUN/CCND1/CASP3/AKT1 | |
| hsa04657 | IL-17 signaling pathway | 6 | RELA/PTGS2/MAPK8/JUN/HSP90AA1/CASP3 | |
| hsa04625 | C-type lectin receptor signaling pathway | 6 | STAT1/RELA/PTGS2/MAPK8/JUN/AKT1 | |
| hsa04659 | Th17 cell differentiation | 6 | STAT1/RELA/MAPK8/JUN/HSP90AA1/AHR | |
| hsa04380 | Osteoclast differentiation | 6 | STAT1/RELA/PPARG/MAPK8/JUN/AKT1 | |
| hsa05212 | Pancreatic cancer | 5 | STAT1/RELA/MAPK8/CCND1/AKT1 | |
| hsa05161 | Hepatitis B | 6 | STAT1/RELA/MAPK8/JUN/CASP3/AKT1 | |
| hsa05210 | Colorectal cancer | 5 | MAPK8/JUN/CCND1/CASP3/AKT1 | |
| hsa05222 | Small cell lung cancer | 5 | RELA/PTGS2/CCND1/CASP3/AKT1 | |
| hsa01522 | Endocrine resistance | 5 | MAPK8/JUN/ESR1/CCND1/AKT1 | |
| hsa04620 | Toll-like receptor signaling pathway | 5 | STAT1/RELA/MAPK8/JUN/AKT1 | |
| hsa05145 | Toxoplasmosis | 5 | STAT1/RELA/MAPK8/CASP3/AKT1 | |
| hsa05166 | Human T-cell leukemia virus 1 infection | 6 | RELA/MAPK8/JUN/ICAM1/CCND1/AKT1 | |
| hsa04926 | Relaxin signaling pathway | 5 | RELA/NOS3/MAPK8/JUN/AKT1 | |
